# Supplementary material for: The Physical Activity and Nutritional INfluences in Ageing (PANINI) Toolkit: A Standardized Approach towards Physical Activity and Nutritional Assessment of Older Adults
Source: Healthcare (Basel). 2022 May 31;10(6):1017. doi: 10.3390/healthcare10061017 (PMC9222478; doi:10.3390/healthcare10061017)
Supplement: Supplementary file 1 [file healthcare-10-01017-s001.zip › PANINI CRF.pdf]

PANINI Case Report Form

Contents

Patient history form....3

Mini Nutritional Assessment (MNA®)....4

Height and weight....5

Dual-energy X-ray absorptiometry....5

Bioelectrical Impedance Analysis (BIA)....5

Circumferences....6

Handgrip strength....7

Accelerometry....7

Short Physical Performance Battery (SPPB, Guralnik, J. M et al., 1994)....8

Balance tests eyes closed....11

Modified Minnesota Leisure Time Activities (MLTA) Questionnaire (Taylor, H. L et al., 1978)....12

The first Fried criteria for frailty....12

Fried criteria for frailty (Fried et al., 2001)....13

Dietary intake....14

Standardised Mini-Mental State Examination (SMMSE)....15

Mini Mental State Examination (MMSE) –page for participant....16

Self-administered Tests....17

Socio-demographic questions....18

Medications information....26

International Physical Activity Questionnaire Short Form (IPAQ, Both, M. L et al., 1999)....27

Short Fall Efficacy Scale International (FES-I, Kempen, G. I et al., 2008)....29

Katz Index of Independence in Activities of Daily Living (Katz ADL, Katz, 1983)....30

Geriatric Depression Scale (GDS-15, Sheikh & Yesavage 1986)....31

Center for Epidemiologic Studies Depression Scale (CES-D) (adapted)....31

Food Frequency Questionnaire (EPIC, 1997)....32

Self-administered questionnaires- information....43

Additional question for water consumption....43

Patient history form

Ask participant if he/she has or has ever had:

- ☐ Diabetes type 1
- ☐ Diabetes type 2
- ☐ High blood pressure
- ☐ High cholesterol
- ☐ Cancer (type) __________________
- ☐ Chronic Obstructive Pulmonary Disease
- ☐ Pulmonary embolism
- ☐ Asthma
- ☐ Atherosclerosis
- ☐ Angina pectoris
- ☐ Cardiovascular disease
- ☐ Myocardial infarction
- ☐ Stroke
- ☐ Transient ischemic attack (mini stroke)
- ☐ Epilepsy
- ☐ Parkinson’s disease
- ☐ Cataracts
- ☐ Kidney failure
- ☐ Osteoarthritis
- ☐ Rheumatoid arthritis
- ☐ Hypothyroidism
- ☐ Hyperthyroidism
- ☐ Depression
- ☐ Dementia (type) __________________

Other medical conditions (please list):

____

____

Mini Nutritional Assessment (MNA®)

Complete the questionnaire by filling the boxes with the appropriate numbers.

Screening

A Has food intake declined over the past 3 months due to loss of appetite, digestive problems, chewing or swallowing difficulties?
0 = severe decrease in food intake
1 = moderate decrease in food intake
2 = no decrease in food intake ☐

B Weight loss during the last 3 months
0 = weight loss greater than 3kg (6.6lbs)
1 = does not know
2 = weight loss between 1 and 3kg (2.2 and 6.6 lbs)
3 = no weight loss ☐

C Mobility
0 = bed or chair bound
1 = able to get out of bed / chair but does not go out
2 = goes out ☐

D Has suffered psychological stress or acute disease in the past 3 months?
0 = yes 2 = no ☐

E Neuropsychological problems
0 = severe dementia or depression
1 = mild dementia
2 = no psychological problems ☐

F Body Mass Index (BMI) = weight in kg / (height in m)^2 ☐
0 = BMI less than 19
1 = BMI 19 to less than 21
2 = BMI 21 to less than 23
3 = BMI 23 or greater ☐

Screening score (subtotal max. 14 points) ☐☐
12-14 points: ☐ Normal nutritional status
8-11 points: ☐ At risk of malnutrition
0-7 points: ☐ Malnourished
For a more in-depth assessment, continue with questions G-R

Assessment

G Lives independently (not in nursing home or hospital)
1 = yes 0 = no ☐

H Takes more than 3 prescription drugs per day
0 = yes 1 = no ☐

I Pressure sores or skin ulcers
0 = yes 1 = no ☐

J How many full meals does the patient eat daily?
0 = 1 meal
1 = 2 meals
2 = 3 meals ☐

K Selected consumption markers for protein intake
- At least one serving of dairy products (milk, cheese, yoghurt) per day yes ☐ no ☐
- Two or more servings of legumes or eggs per week yes ☐ no ☐
- Meat, fish or poultry every day yes ☐ no ☐

0.0 = if 0 or 1 yes
0.5 = if 2 yes
1.0 = if 3 yes ☐.☐

L Consumes two or more servings of fruit or vegetables per day?
0 = no 1 = yes ☐

M How much fluid (water, juice, coffee, tea, milk...) is consumed per day?
0.0 = less than 3 cups
0.5 = 3 to 5 cups
1.0 = more than 5 cups ☐.☐

N Mode of feeding
0 = unable to eat without assistance
1 = self-fed with some difficulty
2 = self-fed without any problem ☐

O Self view of nutritional status
0 = views self as being malnourished
1 = is uncertain of nutritional state
2 = views self as having no nutritional problem ☐

P In comparison with other people of the same age, how does the patient consider his / her health status?
0.0 = not as good
0.5 = does not know
1.0 = as good
2.0 = better ☐.☐

Q Mid-arm circumference (MAC) in cm
0.0 = MAC less than 21
0.5 = MAC 21 to 22
1.0 = MAC greater than 22 ☐.☐

R Calf circumference (CC) in cm
0 = CC less than 31
1 = CC 31 or greater ☐

Assessment (max. 16 points) ☐☐.☐
Screening score ☐☐.☐
Total Assessment (max. 30 points) ☐☐.☐

Malnutrition Indicator Score
24 to 30 points ☐ Normal nutritional status
17 to 23.5 points ☐ At risk of malnutrition
Less than 17 points ☐ Malnourished

References
1. Vellas B, Villars H, Abellan G, *et al.* Overview of the MNA® - Its History and Challenges. *J Nutr Health Aging.* 2006; **10**:456-465.
2. Rubenstein LZ, Harker JO, Salva A, Guigoz Y, Vellas B. Screening for Undernutrition in Geriatric Practice: Developing the Short-Form Mini Nutritional Assessment (MNA-SF). *J. Geront.* 2001; **56A**: M366-377
3. Guigoz Y. The Mini-Nutritional Assessment (MNA®) Review of the Literature - What does it tell us? *J Nutr Health Aging.* 2006; **10**:466-487.

® Société des Produits Nestlé, S.A., Vevey, Switzerland, Trademark Owners
© Nestlé, 1994, Revision 2009. N67200 12/99 10M

For more information: www.mna-elderly.com

Save Print Reset

Nestlé NutritionInstitute

Height and weight

Let the participant take off their shoes and heavy clothes (vest, coat, sweater). Let the participant empty their pockets (wallet, keys, etc.).

Note: Do not correct the weight for the clothes

Height (without shoes)	☐☐☐.☐ cm
Pacemaker?	☐ Yes → weighing scale<br>☐ No → BIA + weighing scale
Weight (without correction for clothes)	Weighing scale: ☐☐☐.☐ kg
Was the measurement possible?	☐ Yes<br>☐ No, reason ____________________________

Dual-energy X-ray absorptiometry

Performed<br>Yes ☐ No ☐	Model of DXA ______________________________
Total mass ☐☐☐,☐ kg<br>Total fat-free soft tissue mass ☐☐,☐ kg<br>Total fat mass ☐☐,☐ kg	Total bone mineral content ☐☐,☐ kg<br>T score ☐,☐

Bioelectrical Impedance Analysis (BIA)

Let the participant take off their shoes and heavy clothes (vest, coat, sweater). Let the participant empty their pockets (wallet, keys, etc.). Let the patient also take off socks, panty and compression stockings. The feet must be fully exposed.

Note: Check if the patient has a pacemaker. Do not perform the BIA measurement if the patient has a pacemaker

Pacemaker?	☐ Yes→ **NO BIA!!!** ☐ No
Alcohol consumption in last 8 h?	☐ Yes ☐ No

Time BIA measurement<br>Time last meal<br>Time last drink<br>Time last visit toilet	☐☐:☐☐ hh:mm (write hours in 0-24 format)<br>☐☐:☐☐ hh:mm<br>☐☐:☐☐ hh:mm<br>☐☐:☐☐ hh:mm
Weight	BIA: ☐☐☐.☐ kg
BMI (calculated by BIA)	☐☐.☐ kg/m^2
Muscle mass	Skeletal muscle mass: ☐☐.☐ kg<br>SMI: ☐☐.☐☐ kg/m^2<br>(skeletal muscle mass (kg)/height $(m)^2$)
Fat mass percentage	☐☐.☐ %
Was the BIA measurement possible?	☐ Yes<br>☐ No, reason ____

Circumferences	
**Put on anti-slip socks.**<br>If not possible (e.g. orthosis, orthopaedic shoes) , give an explanation:<br>____	
Waist circumference	☐☐☐,☐ cm
Hip circumference	☐☐☐, ☐ cm
Calf circumference	☐☐,☐ cm right ☐☐,☐ cm left
Mid-arm circumference	☐☐,☐ cm
Were the measurements possible?	☐ Yes<br>☐ No:<br>Which one: ___________________________________<br>Reason:______________________________________

Handgrip strength	
Dominant hand	Right ☐ Left ☐
Adjust the dynamometer. Arms straight and parallel to the body. 3x Maximal effort on the right and left alternately. **Encourage with "harder, harder, harder"**<br><br>Right 1st trial ☐☐.☐ kg<br>2nd trial ☐☐.☐ kg<br>3rd trial ☐☐.☐ kg	Left 1st trial ☐☐.☐ kg<br>2nd trial ☐☐.☐ kg<br>3rd trial ☐☐.☐ kg
Maximal value right ☐☐.☐ kg	Maximal value left ☐☐.☐ kg
Was the measurement possible?	☐ Yes<br>☐ No, reason ____________________________
Note<br>(e.g. arthrosis which has influenced the measurement)	____

Accelerometry	
Was accelerometry performed?<br>☐ Yes<br>☐ No	If yes, for how many days?<br>☐ days<br>Model of accelerometer:<br>____<br>Where was the accelerometer positioned?<br>☐ Waist<br>☐ Back<br>☐ Ankle<br>☐ Wrist

Short Physical Performance Battery (SPPB, Guralnik, J. M et al., 1994)

Balance tests

Note: Perform the balance tests at the designated location in the room and standardize in every room. Let the patient choose a preferred foot for the semi-tandem and tandem stand. After this the patient cannot switch so the preferred foot stays the same. **Give the following instruction: "stand as still as possible with the arms along the body".** Try a maximum of 3 trials if the patient is not able to maintain for 10 seconds.

	Score		Number of trials (max of 3)
Side-by-side stand 10s<br><br>Feet together side-by-side	Held for 10s<br>Not held for 10s<br>Not attempted	☐ 1 point<br>☐ 0 points<br>☐ 0 points	☐
Semi-tandem stand 10s<br><br>Heel of one foot against side of big toe of the other	Held for 10s<br>Not held for 10s<br>Not attempted	☐ 1 point<br>☐ 0 points<br>☐ 0 points	☐
Tandem stand 10s<br><br>Feet aligned heel to toe	Held for 10s<br>Not held 3 – 9.99s<br>Held for < 3s<br>Not attempted	☐ 2 points<br>☐ 1 point<br>☐ 0 points<br>☐ 0 points	☐

Was the measurement possible?	☐ Yes<br>☐ No, reason ______________________________

4 meter walk

Note: Perform the walk test preferable without the use of walking aid. Give the participant the instruction to walk at their usual pace. Stand over the 4-meter line and start and stop the measurement when the whole foot lands behind the corresponding line on the ground. When the participant is very instable, then walk diagonally behind the participant

	Time (s)	Score
**Start:** 0 meter (directly after the line)<br>**Stop:** 4 meter, walk through to approx. 5 meter to prevent slowing down before the end of the course<br><br>First measurement:<br><br>Second measurement:	☐.☐☐ s<br><br>☐.☐☐ s	Please report both times, but use the best of the 2 to score.<br><br>Time > 8.70s ☐ 1 point<br>Time 6.21 – 8.70s ☐ 2 points<br>Time 4.82 – 6.20s ☐ 3 points<br>Time < 4.82s ☐ 4 points<br>Not able to walk ☐ 0 points

Was the measurement possible?	☐ Yes<br>☐ No, reason ____________________
Can the patient walk without a walking aid?	☐ Yes<br>☐ No
If no: With which hand was the walking aid held?	☐ Right ☐ Left ☐ Both
If no: Which walking aid was used?	☐ Rollator ☐ Walking stick<br>☐ Other: ______________________________
Gait speed in meters per second, based on the fastest time (4/time)	☐.☐☐ m/s

<u>Chair stand test</u>

Pre-test: Participant fold their arms across their chest and try to stand up once from a chair.

	Time (s)	Score	
Measures the time required to perform 5 rises from a chair to an upright position as fast as possible without the use of the arms.<br><br>5 rises:<br>• Arms crossed on chest<br>• Knees bent 90° by adjusting the piano chair.<br>• Straighten the pelvis<br>• Start: from sitting, say "start" and start the time<br>• Count out loud the number of sits<br>• Stop: stand 5th time	☐☐.☐☐ s	Used hands to get up<br><br>Not completed in < 60s<br><br>Time ≥ 16.70s<br><br>Time 13.70 – 16.69s<br><br>Time 11.20 – 13.69s<br><br>Time ≤ 11.19s	☐ 0 points<br><br>☐ 0 points<br><br>☐ 1 point<br><br>☐ 2 points<br><br>☐ 3 points<br><br>☐ 4 points

Was the measurement possible?	☐ Yes<br><br>☐ No, reason:____________________________
Used hands to get up?	☐ Yes<br><br>☐ No

Total SPPB score: ☐☐/12

Balance tests eyes closed

Note: Perform these balance tests in the same way as the balance test in the SPPB protocol, but with the only difference that the eyes are closed

	**Able to maintain for 10s**	**Seconds if not able to maintain for 10s**	**Number of trials (max of 3)**
Side-by-side stand 10s<br><br>Feet together side-by-side	☐ Yes<br>☐ No	☐,☐☐ s	☐
Semi-tandem stand 10s<br><br>Heel of one foot against side of big toe of the other	☐ Yes<br>☐ No	☐,☐☐ s	☐
Tandem stand 10s<br><br>Feet aligned heel to toe	☐ Yes<br>☐ No	☐,☐☐ s	☐

Was the measurement possible?	☐ Yes<br>☐ No, reason:___________________________

Modified Minnesota Leisure Time Activities (MLTA) Questionnaire (Taylor, H. L et al., 1978)

Tell to the participant:

I am going to read a list of activities. Please tell me which activities you have done in the past two weeks.

I am going to read a list of activities. Please tell me which activities you have done in the past two weeks:			How often have you name the activity in the last two weeks?	What is the average amount of the time that you spent per session?		How many months per year do you name of activity?
	YES	NO	#times	# hours	#minutes	# months
Walking for exercise?	☐	☐	☐☐	☐☐	☐☐	☐☐
Moderately strenuous household chores, for example scrubbing or vacuuming?	☐	☐	☐☐	☐☐	☐☐	☐☐
Mowing the lawn?	☐	☐	☐☐	☐☐	☐☐	☐☐
Calisthenics/general exercising?	☐	☐	☐☐	☐☐	☐☐	☐☐
Gardening?	☐	☐	☐☐	☐☐	☐☐	☐☐
Golfing?	☐	☐	☐☐	☐☐	☐☐	☐☐

The first Fried criteria for frailty

Did a participant have an unintentional loss of weight in previous year?	☐ YES ☐ NO
If yes, how much?	☐☐, ☐ kg
Is unintentional loss of weight was more or equal to 4.5 kg or at least 5% of the previous year's body weight?	☐ YES ☐ NO

Fried criteria for frailty (Fried et al., 2001)

	Criteria
1. Shrinking, i.e. weight loss	Unintentional loss of 4.5 kg in the year before the current evaluation or unintentional weight loss of at least 5% of the previous year's body weight
2. Weakness, i.e. low handgrip strength	Grip strength of the dominant hand (mean of 3 measurements), using a Jamar hand-held dynamometer:<br><br>BMI/male – Cutoff (kg) – BMI/female – Cutoff (kg)<br>≤24 – ≤29 – ≤23 – ≤17<br>24-26 – ≤30 – 23-26 – ≤17.3<br>26-28 – ≤30 – 26-29 – ≤18<br>>28 – ≤32 – >29 – ≤21
3. Poor endurance, i.e. self-reported exhaustion	Evaluation of two statements of the CES-D scale (in the self-administered part):<br>(a) I felt that everything I did was an effort<br>(b) I could not get going<br>Criterion positive if at least one condition is present for 3 days or more during the last week.
4. Slowness, i.e. low gait speed	Cutoff for time to walk 4 m at usual pace (static protocol):<br><br>Height/male (cm) – Cutoff (m/s) – Height/female (cm) – Cutoff (m/s)<br>≤173 – ≥0.65 m/s – ≤159 – ≥0.65 m/s<br>>173 – ≥0.76 m/s – >159 – ≥0.76 m/s
5. Low activity, i.e. reduced energy consumption	Evaluation of the Modified Minnesota Leisure Time Physical Activity Questionnaire.

Criteria	
1. Shrinking	☐ Yes ☐ No
2. Weakness	☐ Yes ☐ No
3. Poor endurance	☐ Yes ☐ No
4. Slowness	☐ Yes ☐ No
5. Low activity	☐ Yes ☐ No
Score:	☐ out of 5
Result:	• Frail: 3 or more criteria ☐<br>• Prefrail: 1 or 2 criteria ☐<br>• Not frail: 0 criteria ☐

Dietary intake

Is the food frequency questionnaire completed?	☐ Yes<br>☐ No, reason ______________________

Notes for food frequency questionnaire:

____

____

Macronutrients

Energy: ☐☐☐☐ kcal

Fat: ☐☐,☐ g ☐☐,☐ en%

Saturated fat: ☐☐,☐ g ☐☐,☐ en%

Carbohydrates: ☐☐☐,☐ g ☐☐,☐ en%

Protein: ☐☐☐,☐ g ☐,☐ g/kg/body weight

Fibres: ☐☐,☐ g Salt ☐☐,☐ g

Minerals

Sodium: ☐☐☐☐ mg Potassium: ☐☐☐☐ mg

Calcium: ☐☐☐☐ mg Magnesium: ☐☐☐☐ mg

Iron: ☐☐,☐ mg Selenium: ☐☐ µg

Zinc: ☐☐,☐ mg Iodine: ☐☐☐ µg

Vitamins

Vitamin A: ☐☐☐☐ µg Vitamin D: ☐,☐ µg

Vitamin E: ☐☐,☐ mg Vitamin B1: ☐,☐☐ mg

Vitamin B2: ☐,☐☐ mg Vitamin B6: ☐,☐☐ mg

Folic acid: ☐☐☐ µg Vitamin B12: ☐☐,☐☐ µg

Vitamin B3: ☐☐,☐ mg Vitamin C: ☐☐☐ mg

Drinking fluids: ☐☐☐☐ ml

Standardised Mini-Mental State Examination (SMMSE)

Please see accompanying guide for directions for administration

Say: I am going to ask you some questions and give you some problems to solve. Please try to answer as best you can.

1.	(Allow 10 seconds for each reply). **Say**:	
	a) What year is this? (Accept exact answer only)	/1
	b) What season is this? (During the last week of the old season or first week of a new season, accept either)	/1
	c) What month is this? (On the first day of a new month or the last day of the previous month, accept either)	/1
	d) What is today's date? (Accept previous or next date)	/1
	e) What day of the week is this? (Accept exact answer only)	/1
2.	(Allow 10 seconds for each reply). **Say**:	
	a) What country are we in? (Accept exact answer only)	/1
	b) What county are we in? (Accept exact answer only)	/1
	c) What city/town are we in? (Accept exact answer only)	/1
	d) (At home) What is the street address of this house? (Accept street name and house number or equivalent in rural areas)<br>(In facility) What is the name of this building? (Accept exact name of institution only	/1
	e) (At home) What room are we in? (Accept exact answer only)<br>(In facility) What floor of the building are we on? (Accept exact answer only)	/1
3.	**Say**: I am going to name three objects. When I am finished, I want you to repeat them. Remember what they are because I am going to ask you to name them again in a few minutes (Say slowly at approximately one-second intervals).<br>**Ball** **Car** **Man**<br>For repeated use: Bell, jar, fan; bill, tar, can; bull, bar, pan<br>**Say**: Please repeat the three items for me. (Score one point for each correct reply on the first attempt Allow 20 seconds for reply; if the person did not repeat all three, repeat until they are learned or up to a maximum of five times. (But only score first attempt).	/3
4.	**Spell the word WORLD**. (You may help the person to spell the word correctly) **Say**: Now spell it backwards please (Allow 30 seconds; if the subject cannot spell World even with assistance, score 0) Refer to accompanying guide for scoring instructions	/5
5.	**Say**: Now what were the three objects I asked you to remember? (Score one point for each correct answer regardless of order; allow 10 seconds)	/3
6.	Show wristwatch. **Ask:** *What is this called?*<br>Score one point for correct response; accept "pencil" only; score 0 for pen; allow 10 seconds for reply)	/1
7.	Show pencil. **Ask:** *What is this called?*<br>(Score one point for correct response; accept "pencil" only; score 0 for pen; allow 10 seconds for reply)	/1
8.	**Say**: I would like you to repeat a phrase after me: *No ifs, ands, or buts*<br>(Allow 10 seconds for response. Score one point for a correct repetition. Must be exact, e.g. no ifs or buts, score 0)	/1
9.	**Say**: *Read the words on this page and then do what it says*<br>Then, **hand** the person the sheet with CLOSE YOUR EYES (score on reverse of this sheet) on it. If the subject just reads and does not close eyes, you may repeat: Read the words on this page and then do what it says, (a maximum of three times. See point No. 3 in Directions for Administration section of accompanying guide). Allow 10 seconds, score one point only if the subject closes eyes. The subject does not have to read aloud.	/1
10.	**Hand** the person a pencil and paper. **Say:** *Write any complete sentence on that piece of paper.* (Allow 30 seconds. Score one point. The sentence must make sense. Ignore spelling errors).	/1
11.	**Place** design (see reverse of this sheet), pencil, eraser and paper in front of the person. **Say:** *Copy this design please*. Allow multiple tries. Wait until the person is finished and hands it back. Score one point for a correctly copied diagram. The person must have drawn a four-sided figure between two five-sided figures. Maximum time: One minute	/1
12.	**Ask** the person if he is right or left handed. **Take** a piece of paper, hold it up in front of the person and **say** the following: *Take this paper in your right/left hand (whichever is non-dominant), fold the paper in half once with both hands and put the paper down on the floor.*	

Takes paper in correct hand	/1
Folds it in half	/1
Puts it on the floor	/1
**TOTAL TEST SCORE:**	/30
ADJUSTED SCORE	/

Molloy DW, Alemayehu E, Roberts R. Reliability of a standardized Mini-Mental State Examination compared with the traditional Mini-Mental state Examination. *American Journal of Psychiatry*, Vol. 14, 1991a, pp.102-105. The Standardised Mini-Mental State Examination (SMMSE) is the copyright of Dr D.W. Molloy and may not be reproduced without the written consent of the author.

Close your eyes

10.____

____

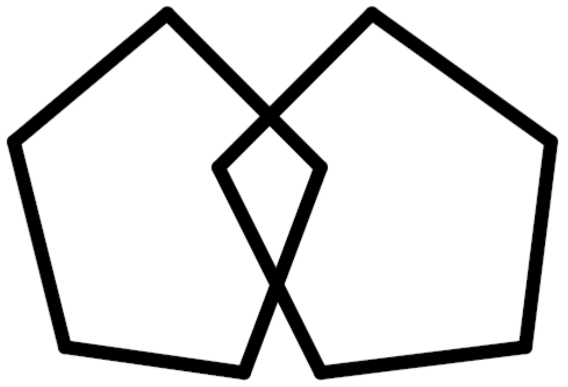

11.____

Time: …………………………………

PANINI Case Report Form

Self-administered Tests

1. Socio-demographic questions	
1.1 What is your country of origin?	___________________________
1.2 Do you speak a language other than English at home?	1. ☐ No, English only<br>2. ☐ Yes, please specify<br>___________________________
1.3 What is your current marital status?	1. ☐ Never married<br>2. ☐ Married, since ☐☐☐☐ (year)<br>3. ☐ Separated/ Divorced, since ☐☐☐☐ (year)<br>4. ☐ Widowed, since ☐☐☐☐ (year)
**2. Education**	
2.1 What is the highest educational qualification you have completed?	1. ☐ Primary education<br>2. ☐ Secondary education<br>3. ☐ Degree or diploma from a university<br>4. ☐ Post graduate degree
2.2 How many years did you go to school?<br>(Counted from the beginning of primary school)	Total school years: ☐☐
2.3 What was your main occupation before you retired?	___________________________

3. Social	
3.1 Who do you live with?<br>(Please choose all that apply.)	1. ☐ No one/ live alone<br>2. ☐ Partner/ spouse<br>3. ☐ Sibling(s) e.g. brother, sister<br>4. ☐ One or more children<br>5. ☐ Other, please specify<br>____
3.2 Where do you live?	1. ☐ Home<br>a. ☐ Privately owned<br>b. ☐ Private rental<br>c. ☐ Public rental<br>d. ☐ Someone's else home<br>2. ☐ In aged care facility<br>3. ☐ In a serviced apartment / retirement village
3.3 Do you have a pet(s)?	1. ☐ No<br>2. ☐ Yes<br>a. ☐ Dog<br>b. ☐ Cat<br>c. ☐ Bird<br>d. ☐ Others, please specify<br>____<br>____

4. Alcohol, smoking, drugs	
4.1 Do you currently drink alcohol?	1. ☐ No<br>2. ☐ Yes, (go to question 4.3)<br>a. ☐ Beer, quantity: _______ /week<br>b. ☐ Wine, quantity: _______ /week<br>c. ☐ Spirit, quantity: _______ /week<br>d. ☐ Others, please specify _______________________<br>quantity: __________ /week
4.2 Have you ever consumed alcohol?	1. ☐ No<br>2. ☐ Yes<br>since: ☐☐☐☐ (year)<br>until: ☐☐☐☐ (year)<br>Type: ____________________, quantity: ______per week
4.3 How many times a week do you drink alcohol in general?	1. ☐ Daily<br>2. ☐ Only on certain days of the week<br>3. ☐ On special occasions
4.4 At what time of the day do you usually drink alcohol?	1. ☐ Morning<br>2. ☐ Afternoon<br>3. ☐ Evening
4.5 Do you currently smoke?	1. ☐ No<br>2. ☐ Yes: (go to question 4.7)<br>a. ☐ cigarettes<br>quantity: ☐☐ cigarettes/day<br>since: ☐☐☐☐ (year)<br>b. ☐ cigar<br>c. ☐ pipe

4.6 Have you ever smoked?	1. ☐ No<br>2. ☐ Yes<br>since: ☐☐☐☐ (year)<br>until: ☐☐☐☐ (year)<br>quantity: ☐☐ cigarettes/day
4.7 Do you use any illicit drugs?	1. ☐ No<br>2. ☐ Yes, please specify<br>____

5 Mobility	
Please consider your situation over the last 3 months.	
5.1 Do you have any problems with walking? (Types of problems you may have affecting your mobility could include shortness of breath, or pain, etc.)	1. ☐ No<br>2. ☐ Yes
5.2 Do you need something/someone to help you move around *indoors*?<br><br>(Please choose all that apply.)	1. ☐ No<br>2. ☐ Yes,<br>a. ☐ Walking holding someone's arm<br>b. ☐ Walking holding furniture<br>b. ☐ Walking stick<br>c. ☐ Walking Frame (no wheels)<br>d. ☐ Walking Frame (with wheels)<br>e. ☐ Wheelchair<br>f. ☐ Others, please specify ______________________
5.3 Do you need something/someone to help you move around *outdoors*?<br><br>(Please choose all that apply.)	1. ☐ No<br>2. ☐ Yes,<br>a. ☐ Walking holding someone's arm<br>b. ☐ Walking holding furniture<br>b. ☐ Walking stick<br>c. ☐ Walking Frame (no wheels)<br>d. ☐ Walking Frame (with wheels)<br>e. ☐ Wheelchair<br>f. ☐ Others, please specify ______________________
5.4 Do you use a mobility scooter?	1. ☐ No<br>2. ☐ Yes
5.5 Were you immobile or bedridden for longer than 1 week in the past 3 months?	1. ☐ No<br>2. ☐ Yes

5.6 Do you usually go outside every day?	1. ☐ No<br>2. ☐ Yes
5.7 Can you climb a flight of 10 stairs?	1. ☐ Not anymore<br>When did you stop doing that?<br>☐☐ months/years ago (*please circle*)<br>2. ☐ Yes
5.8 Do you cycle?	1. ☐No, never<br>2. ☐ Not anymore<br>When did you stop doing that?<br>☐☐ months/years ago *(please circle)*<br>3. ☐ Yes
5.9 Do you drive?	1. ☐ No, never done it<br>2. ☐ Not anymore<br>When did you stop doing that?<br>☐☐ months/years ago (*please circle*)<br>3. ☐ Yes
5.10 Do you travel on public transport on your own?	1. ☐ No, never<br>2. ☐ Not anymore<br>When did you stop doing that?<br>☐☐ months/years ago *(please circle)*<br>3. ☐ Yes
5.11 What is your highest level of physical fitness?	1. ☐ Gymnastics / sports / gym / fitness<br>2. ☐ Walking outdoors more than 20 minutes without a rest, at least 1 time per week<br>3. ☐ Walking outdoors less than 20 minutes without a rest, at least 1 time per week<br>4. ☐ Walking indoors<br>5. ☐ Wheelchair bound / bedridden

5.12 What is your maximum walking distance?	1. ☐ Over 1 kilometre<br>2. ☐ Between 250 meters and 1 kilometre<br>3. ☐ Less than 250 meters

**6. Falls history**	
6.1 Have you had any falls in the past year?	1. ☐ No<br>2. ☐ Yes, ☐☐ time(s)
6.2 In the past year were there any occasions when you almost fell?	1. ☐ No<br>2. ☐ Yes, ☐☐ time(s)
6.3 Do you have any concern with your standing balance?	1. ☐ Never<br>2. ☐ Sometimes<br>3. ☐ Regularly<br>4. ☐ Always
6.4 Do you get dizzy?	1. ☐ Never<br>2. ☐ Sometimes<br>3. ☐ Regularly<br>4. ☐ Always
6.5 Do you have a personal alarm?	1. ☐ No<br>2. ☐ Yes
6.6 Have you received any recent home modifications to make the environment safer for you?	1. ☐ No<br>2. ☐ Yes
6.7 Do you have any problems with your feet?	1. ☐ Calluses (thickened hard skin)<br>2. ☐ Bunions<br>3. ☐ Ingrown toenails<br>4. ☐ Tingling/ Numbness<br>5. ☐ Pain

6.8 Do you cut your toenails by yourself?	1. ☐ No<br>2. ☐ Yes
6.9 Do you visit a podiatrist regularly for foot care?	1. ☐ No<br>2. ☐ Yes
6.10 What kind of footwear do you usually wear *indoor*?	1. ☐ Sandals<br>2. ☐ Slippers<br>3. ☐ Sport shoes<br>4. ☐ Flats<br>5. ☐ Boots<br>6. ☐ Other, please specify ___________________________
6.11 What kind of footwear do you usually wear *outdoor*?	1. ☐ Sandals<br>2. ☐ Sport shoes<br>3. ☐ Flats<br>4. ☐ Boots<br>5. ☐ Other, please specify ____________________________
6.12 What kind of footwear did you wear when you last fell?	1. ☐ Sandals<br>2. ☐ Slippers<br>3. ☐ Sport shoes<br>4. ☐ Flats<br>5. ☐ Boots<br>6. ☐ Other, please specify ____________________________

Medications information

Are you currently taking any medications (prescription, over the counter, vitamins, minerals, supplements), or non-drug therapy?							
#	Medication/ Non-drug Therapy	Indication	Dose (per admin)	Dose Units[1]	Frequency[2]	Route of Administration[3]	Taken regularly
							☐ YES ☐ NO
							☐ YES ☐ NO
							☐ YES ☐ NO
							☐ YES ☐ NO
							☐ YES ☐ NO
							☐ YES ☐ NO
							☐ YES ☐ NO
							☐ YES ☐ NO
							☐ YES ☐ NO
							☐ YES ☐ NO

Dose Units[1]

1 - g (gram)
2 - mg (milligram)
3 - µg (microgram)
4 - L (liter)
5 - mL (milliliter)
6 - IU (International Unit)
7 - Other

Frequency[2]

1 - QD (once a day)
2 - BID (twice a day)
3 - TID (three times a day)
4 - QID (four times a day)
5 - QOD (every other day)
6 - QM (every month)
7 - QOM (every other month)
8 - QH (every hour)
9 - AC (before meals)
10 - PC (after meals)
11 - PRN (as needed)
12 - Other

Route of Administration[3]

1 - Oral
2 - Topical
3 - Subcutaneous
4 - Intradermal
5 - Transdermal
6 - Intraocular
7 - Intramuscular
8 - Inhalation
9 - Intravenous
10 - Intraperitoneal
11 - Nasal
12 - Vaginal
13 - Rectal
14 - Other

International Physical Activity Questionnaire Short Form (IPAQ, Both, M. L et al., 1999)

We are interested in finding out about the kinds of physical activities that people do as part of their everyday lives. The questions will ask you about the time you spent being physically active in the **last 7 days**. Please answer each question even if you do not consider yourself to be an active person. Please think about the activities you do at work, as part of your house and yard work, to get from place to place, and in your spare time for recreation, exercise or sport.

Think about all the **vigorous** activities that you did in the **last 7 days**. **Vigorous** physical activities refer to activities that take hard physical effort and make you breathe much harder than normal. Think *only* about those physical activities that you did for at least 10 minutes at a time.

1. During the **last 7 days**, on how many days did you do **vigorous** physical activities like heavy lifting, digging, aerobics, or fast bicycling?

   _____ **days per week**

   ☐ No vigorous physical activities → ***Skip to question 3***

2. How much time did you usually spend doing **vigorous** physical activities on one of those days?

   _____ **hours per day**

   _____ **minutes per day**

   ☐ Don't know/Not sure

Think about all the **moderate** activities that you did in the **last 7 days**. **Moderate** activities refer to activities that take moderate physical effort and make you breathe somewhat harder than normal. Think only about those physical activities that you did for at least 10 minutes at a time.

3. During the **last 7 days**, on how many days did you do **moderate** physical activities like carrying light loads, bicycling at a regular pace, or doubles tennis? Do not include walking.

   _____ **days per week**

   ☐ No moderate physical activities → ***Skip to question 5***

4. How much time did you usually spend doing **moderate** physical activities on one of those days?

   _____ **hours per day**

_____ **minutes per day**

☐ Don't know/Not sure

Think about the time you spent **walking** in the **last 7 days**. This includes at work and at home, walking to travel from place to place, and any other walking that you have done solely for recreation, sport, exercise, or leisure.

5. During the **last 7 days**, on how many days did you **walk** for at least 10 minutes at a time?

_____ **days per week**

☐ No walking → ***Skip to question 7***

6. How much time did you usually spend **walking** on one of those days?

_____ **hours per day**

_____ **minutes per day**

☐ Don't know/Not sure

The last question is about the time you spent **sitting** on weekdays during the **last 7 days**. Include time spent at work, at home, while doing course work and during leisure time. This may include time spent sitting at a desk, visiting friends, reading, or sitting or lying down to watch television.

7. During the **last 7 days**, how much time did you spend **sitting** on a **week day**?

_____ **hours per day**

_____ **minutes per day**

☐ Don't know/Not sure

Short Fall Efficacy Scale International (FES-I, Kempen, G. I et al., 2008)

Now we would like to ask some questions about how concerned you are about the possibility of falling. Please reply thinking about how you usually do the activity. If you currently do not do the activity, please answer to show whether you think you would be concerned about falling IF you did the activity. For each of the following activities, please tick the box which is closest to your own opinion to show how concerned you are that you might fall IF you did this activity.

		Not at all concerned 1	Somewhat concerned 2	Fairly concerned 3	Very concerned 4
1	Getting dressed or undressed	☐ 1	☐ 2	☐ 3	☐ 4
2	Taking a bath or shower	☐ 1	☐ 2	☐ 3	☐ 4
3	Getting in or out of a chair	☐ 1	☐ 2	☐ 3	☐ 4
4	Going up or down stairs	☐ 1	☐ 2	☐ 3	☐ 4
5	Reaching for something above your head or on the ground	☐ 1	☐ 2	☐ 3	☐ 4
6	Walking up or down a slope	☐ 1	☐ 2	☐ 3	☐ 4
7	Going out to a social event (e.g. religious service, family gathering or club meeting)	☐ 1	☐ 2	☐ 3	☐ 4

Katz Index of Independence in Activities of Daily Living (Katz ADL, Katz, 1983)

Activity	Independent
	ANSWER
1. Bathing (sponge bath, tub bath, or shower)<br>Receives either no assistance or assistance in bathing only one part of body	☐ YES ☐ NO
2. Dressing<br>Gets clothes and dresses without any assistance except for tying shoes	☐ YES ☐ NO
3. Toileting<br>Goes to toilet room, uses toilet, arranges clothes, and returns without any assistance (may use cane or walker for support and may use bedpan/urinal at night	☐ YES ☐ NO
4. Transferring<br>Moves in and out of bed and chair without assistance (may use can or walker)	☐ YES ☐ NO
5. Continence<br>Controls bowel and bladder completely by self (without occasional "accidents")	☐ YES ☐ NO
6. Feeding<br>Feeds self without assistance (except for help with cutting meat or buttering bread)	☐ YES ☐ NO

Geriatric Depression Scale (GDS-15, Sheikh & Yesavage 1986)

Choose the best answer for how you felt **over the past week**:

Question	Answer	Score
1. Are you basically satisfied with your life?	☐ YES ☐ NO	
2. Have you dropped many of your activities and interests?	☐ YES ☐ NO	
3. Do you feel that your life is empty?	☐ YES ☐ NO	
4. Do you often get bored?	☐ YES ☐ NO	
5. Are you in good spirits most of the time?	☐ YES ☐ NO	
6. Are you afraid that something bad is going to happen to you?	☐ YES ☐ NO	
7. Do you feel happy most of the time?	☐ YES ☐ NO	
8. Do you often feel helpless	☐ YES ☐ NO	
9. Do you prefer to stay at home, rather than going out and doing new things?	☐ YES ☐ NO	
10. Do you feel you have more problems with memory than most?	☐ YES ☐ NO	
11. Do you think it is wonderful to be alive now?	☐ YES ☐ NO	
12. Do you feel pretty worthless the way you are now?	☐ YES ☐ NO	
13. Do you feel full of energy?	☐ YES ☐ NO	
14. Do you feel that your situation is hopeless?	☐ YES ☐ NO	
15. Do you think that most people are better off than you are?	☐ YES ☐ NO	

Center for Epidemiologic Studies Depression Scale (CES-D) (adapted)

Instructions: Below is a list of the ways you might have felt or behaved. Please tell me how often you have felt this way **during the past week**.

	Rarely or none of the time (less than 1 day)	Some or a little of the time 1-2 days)	Occasionally or a moderate amount of time (3-4 days)	Most or all of the time (5-7 days)
I felt that everything I did was an effort				
I could not get going				

Food Frequency Questionnaire (EPIC, 1997)

This questionnaire asks for some background information about you, especially about what you eat.

Please answer every question. If you are uncertain about how to answer a question then do the best you can, but please do not leave a question blank.

1. YOUR DIET LAST YEAR

For each food there is an amount shown, either a "medium serving" or a common household unit such as a slice or teaspoon. Please put a tick (✓) in the box to indicate how often, on average, you have eaten the specified amount of each food **during the past year.**

EXAMPLES

For white bread the amount is one slice, so if you ate 4 or 5 slices a day, you should put a tick in the column headed "4-5 per day".

FOODS AND AMOUNTS	AVERAGE USE LAST YEAR								
BREAD AND SAVOURY BISCUITS (one slice or biscuit)	Never or less than once/month	1-3 per month	Once a week	2-4 per week	5-6 per week	Once a day	2-3 per day	4-5 per day	6+per day
White bread and rolls								✓	

For chips, the amount is a "medium serving", so if you had a helping of chips twice a week you should put a tick in the column headed "2-4 per week".

FOODS AND AMOUNTS	AVERAGE USE LAST YEAR								
POTATOES, RICE AND PASTA (medium serving)	Never or less than once/month	1-3 per month	Once a week	2-4 per week	5-6 per week	Once a day	2-3 per day	4-5 per day	6+per day
Chips				✓					

For very seasonal fruit such as strawberries and raspberries you should estimate your average use when the fruits are in season, so if you ate strawberries or raspberries about once a week when they were in season you should put a tick in the column headed "once a week".

FOODS AND AMOUNTS	AVERAGE USE LAST YEAR								
FRUIT (1 fruit or medium serving)	Never or less than once/month	1-3 per month	Once a week	2-4 per week	5-6 per week	Once a day	2-3 per day	4-5 per day	6+per day
Strawberries, raspberries, kiwi fruit			✓						

Please estimate your average food use as best you can, and please answer every question - do not leave ANY lines blank.

PLEASE PUT A TICK (✓) ON EVERY LINE

FOODS AND AMOUNTS	AVER AGE USE LAST YEAR								
MEAT AND FISH (medium serving)	Never or less than once/month	1-3 per month	Once a week	2-4 per week	5-6 per week	Once a day	2-3 per day	4-5 per day	6+ per day
Beef: roast, steak, mince, stew or casserole									
Beefburgers									
Pork: roast, chops, stew or slices									
Lamb: roast, chops or stew									
Chicken or other poultry e.g. turkey									
Bacon									
Ham									
Corned beef, Spam, luncheon meets									
Sausages									
Savoury pies, e.g. meat pie, pork pie, pasties, steak & kidney pie, sausage rolls									
Liver, liver pâté, liver sausage									
Fried fish in batter, as in fish and chips									
Fish fingers, fish cakes									
Other white fish, fresh or frozen, e.g. cod, haddock, plaice, sole, halibut									
Oily fish, fresh or canned, e.g. mackerel, kippers, tuna, salmon, sardines, herring									
Shellfish, e.g. crab, prawns, mussels									
Fish roe, taramasalata									
	Never or less than once/month	1-3 per month	Once a week	2-4 per week	5-6 per week	Once a day	2-3 per day	4-5 per day	6+ per day

Please check that you have a tick (✓) on EVERY line

PLEASE PUT A TICK (✓) ON EVERY LINE

FOODS AND AMOUNTS	AVERAGE USE LAST YEAR								
BREAD AND SAVOURY BISCUITS (one slice or biscuit)	Never or less than once/month	1-3 per month	Once a week	2-4 per week	5-6 per week	Once a day	2-3 per day	4-5 per day	6+ per day
White bread and rolls									
Brown bread									
Wholemeal bread and rolls									
Cream crackers, cheese biscuits									
Crispbread, e.g. Ryvita									
**CEREALS** (one bowl)									
Porridge, Readybrek									
Breakfast cereal such as cornflakes , muesli etc.									
**POTATOES, RICE AND PASTA** (medium serving)									
Boiled, mashed, instant or jacket potatoes									
Chips									
Roast potatoes									
Potato salad									
White rice									
Brown rice									
White or green pasta, e.g. spaghetti , macaroni, noodles									
Wholemeal pasta									
Lasagne, moussaka									
Pizza									
	Never or less than once/month	1-3 per month	Once a week	2-4 per week	5-6 per week	Once a day	2-3 per day	4-5 per day	6+ per day

Please check that you have a tick (✓) on EVERY line

PLEASE PUT A TICK (✓) ON EVERY LINE

**FOOOS ANO AMOUNTS**	**AVERAGE USE LAST YEAR**								
**DAIRY PROOUCTS AND FATS**	Never or less than once/month	1-3 per month	Once a week	2-4 per week	5-6 per week	Once a day	2-3 per day	4-5 per day	6+ per day
Single or sour cream (tablespoon)									
Double or clotted cream (tablespoon)									
Low fat yogurt, fromage frais (125g carton)									
Full fat or Greek yogurt (125g carton)									
Dairy desserts (125g carton)									
Cheese, e.g. Cheddar, Brie, Edam (medium serving)									
Cottage cheese, low fat soft cheese (medium serving)									
Eggs as boiled, fried, scrambled , etc. (one)									
Quiche (medium serving)									
Low calorie, low fat salad cream(tablespoon)									
Salad cream, mayonnaise (tablespoon)									
French dressing (tablespoon)									
Other salad dressing (tablespoon)									
**The following on bread or vegetables**									
Butter (teaspoon)									
Block margarine, e.g. Stork, Krona (teaspoon)									
Polyunsaturated margarine (tub), e.g. Flora, sunflower (teaspoon)									
Other soft margarine, dairy spreads (tub), e.g. Blue Band, Clover (teaspoon)									
Low fat spread (tub), e.g. Outline, Gold (teaspoon)									
Very low fat spread (tub) (teaspoon)									
	Never or less than once/month	1-3 per month	Once a week	2-4 per week	5-6 per week	Once a day	2-3 per day	4-5 per day	6+ per day

Please check that you have a tick (✓) on EVERY line

PLEASE PUT A TICK (✓) ON EVERY LINE

FOODS AND AMOUNTS	AVERAGE USE LAST YEAR								
**SWEETS AND SNACKS** (medium serving)	Never or less than once/month	1-3 per month	Once a week	2-4 per week	5-6 per week	Once a day	2-3 per day	4-5 per day	6+ per day
Sweet biscuits, chocolate , e.g. digestive (one)									
Sweet biscuits, plain, e.g. Nice, ginger(one)									
Cakes e.g. fruit, sponge , home baked									
Cakes e.g. fruit, sponge, ready made									
Buns, pastries e.g. scones, flapjacks, home baked									
Buns, pastries e.g. croissants, doughnuts, ready made									
Fruit pies, tarts, crumbles, home baked									
Fruit pies, tarts, crumbles, ready made									
Sponge puddings, home baked									
Sponge puddings, ready made									
Milk puddings, e.g. rice, custard, trifle									
Ice cream, choc-ices									
Chocolates, single or squares									
Chocolate snack bars e.g. Mars, Crunchie									
Sweets, toffees, mints									
Sugar added to tea, coffee, cereal (teaspoon)									
Crisps or other packet snacks, e.g. Wotsits									
Peanuts or other nuts									
**SOUPS, SAUCES, AND SPREADS**									
Vegetable soups (bowl)									
Meat soups (bowl)									
Sauces, e.g. white sauce, cheese sauce, gravy (tablespoon)									
Tomato ketchup (tablespoon)									
Pickles, chutney (tablespoon)									
Marmite, Bovril (teaspoon)									
Jam, marmalade , honey (teaspoon)									
Peanut butter (teaspoon)									
	Never or less than once/month	1-3 per month	Once a week	2-4 per week	5-6 per week	Once a day	2-3 per day	4-5 per day	6+ per day

Please check that you have a tick (✓) on EVERY line

PLEASE PUT A TICK (✓) ON EVERY LINE

FOODS AND AMOUNTS	AVERAGE USE LAST YEAR								
**DRINKS**	Never or less than once/month	1-3 per month	Once a week	2-4 per week	5-6 per week	Once a day	2-3 per day	4-5 per day	6+ per day
Tea (cup)									
Coffee, instant or ground (cup)									
Coffee, decaffeinated (cup)									
Coffee whitener, e.g. Coffee-mate (teaspoon)									
Cocoa, hot chocolate (cup)									
Horlicks, Ovaltine (cup)									
Wine (glass)									
Beer, lager or cider (half pint)									
Port, sherry, vermouth, liqueurs (glass)									
Spirits, e.g. gin, brandy, whisky , vodka (single)									
Low calorie or diet fizzy soft drinks (glass)									
Fizzy soft drinks, e.g. Coca cola, lemonade (glass)									
Pure fruit juice (100%) e.g. orange, apple juice (glass)									
Fruit squash or cordial (glass)									
**FRUIT** **For seasonal fruits marked *, please estimate your average use when the fruit is in season**									
Apples (1 fruit)									
Pears (1 fruit)									
Oranges , satsumas , mandarins (1 fruit)									
Grapefruit (half)									
Bananas (1 fruit)									
Grapes (medium serving)									
Melon (1 slice)									
* Peaches, plums, apricots (1 fruit)									
* Strawberries , raspberries, kiwi fruit (medium serving)									
Tinned fruit (medium serving)									
Dried fruit, e.g. raisins, prunes (medium serving)									
	Never or less than once/month	1-3 per month	Once a week	2-4 per week	5-6 per week	Once a day	2-3 per day	4-5 per day	6+ per day

Please check that you have a tick (✓) on EVERY line

PLEASE PUT A TICK (✓) ON EVERY UNE

FOODS AND AMOUNTS	AVERAGE USE LAST YEAR								
**VEGETABLES** Fresh, frozen or tinned (medium serving)	Never or less than once/month	1-3 per month	Once a week	2-4 per week	5-6 per week	Once a day	2-3 per day	4-5 per day	6+ per day
Carrots									
Spinach									
Broccoli, spring greens, kale									
Brussels sprouts									
Cabbage									
Peas									
Green beans, broad beans, runner beans									
Marrow, courgettes									
Cauliflower									
Parsnips, turnips, swedes									
Leeks									
Onions									
Garlic									
Mushrooms									
Sweet peppers									
Beansprouts									
Green salad, lettuce, cucumber, celery									
Watercress									
Tomatoes									
Sweetcorn									
Beetroot									
Coleslaw									
Avocado									
Baked beans									
Dried lentils, beans, peas									
Tofu , soya meat, TVP, Vegeburger									
	Never or less than once/month	1-3 per month	Once a week	2-4 per week	5-6 per week	Once a day	2-3 per day	4-5 per day	6+ per day

Please check that you have a tick (✓) on EVERY line

2. Are there any OTHER foods which you ate more than once a week? ☐ Yes ☐ No
If yes, please list below

Food	Usual serving size	Number of times eaten each week

3. What type of milk did you most often use?
Select one only
Fullcream, silver ☐
Skimmed/ blue ☐
Dried milk ☐
Semi-skimmed, red/white ☐
Channel Islands, gold ☐
Soya ☐
None ☐
Other, specify ____________________________

4. How much milk did you drink each day, including milk with tea, coffee, cereals etc.?
None ☐
Quarter of a pint ☐
Half a pint ☐
Three quarters of a pint ☐
One pint ☐
More than one pint ☐

5. Did you usually eat breakfast cereal (excluding porridge and Ready Brek mentioned earlier)?
☐ Yes ☐ No
If yes, which brand and type of breakfast cereal, including muesli, did you usually eat?
List the one or two types most often used

Brand e.g. Kellogg's ________________________________ Type e.g. cornflakes ____________________________________

________________________________ ____________________________________

6. What kind of fat did you most often use for frying, roasting, grilling etc.?
Select one only
Butter ☐
Lard/ dripping ☐
Vegetable oil ☐
Solid vegetable fat ☐
Margarine ☐
None ☐
If you used vegetable oil, please give type e.g. corn, sunflower _________________________

7. What kind of fat did you most often use for baking cakes etc.?
Select one only
Butter ☐
Lard/ dripping ☐
Vegetable oil ☐
Solid vegetable fat ☐
Margarine ☐
None ☐
If you used vegetable oil, please give name or type e.g. Flora, Stork _________________________

8. How often did you eat food that was fried at home?

Daily ☐ 1-3 times a week ☐ 4-6 times a week ☐
Less than once a week ☐ Never ☐

9. How often did you eat fried food away from home?

Daily ☐ 1-3 times a week ☐ 4-6 times a week ☐
Less than once a week ☐ Never ☐

10. What did you do with the visible fat on your meat?

Ate most of the fat ☐ Ate as little as possible ☐
Ate some of the fat ☐ Did not eat meat ☐

11. How often did you eat grilled or roast meat? ______________ times a week

12. How well cooked did you usually have grilled or roast meat?

Well done /dark brown ☐ Lightly cooked/ rare ☐
Medium ☐ Did not eat meat ☐

13. How often did you add salt to food while cooking?

Always ☐ Rarely ☐
Usually ☐ Never ☐
Sometimes ☐

14. How often did you add salt to food at the table?

Always ☐ Rarely ☐
Usually ☐ Never ☐
Sometimes ☐

15. Did you regularly use a salt substitute (e.g. LoSalt)? ☐ Yes ☐ No

If yes, which brand? ____

16. During the course of last year, on average, how many times a week did you eat the following foods?

**Food type**	**Times/ week**	**Portion size**
Vegetables (not including potatoes)	__________________	Medium serving
Salads	__________________	Medium serving
Fruit and fruit products (not including fruit juice)	__________________	Medium serving or 1 fruit
Fish and fish products	__________________	Medium serving
Meat, meat products and meat dishes (including bacon, ham and chicken)	__________________	Medium serving

17. Have you taken any vitamins, minerals, fish oils, fibre or other food supplements during the past year?

Yes ☐ No ☐ Don't know ☐

If yes, please complete the table below. If you have taken more than 5 types of supplement please put the most frequently consumed brands first.

**Vitamin supplements**	**Average frequency** Tick one box per line to show how often on average you consumed supplements									
**Name and brand** Please list full name, **brand** and strength	Dose Please state number of pills, capsules or teaspoons consumed	Never or less than once a month	1-3 per month	Once a week	2-4 per week	5-6 per week	Once a day	2-3 per day	4-5 per day	6+ per day

Thank you for your help!

Self-administered questionnaires- information

How were the self-administered questionnaires completed?

☐ by participant

☐ by assessor

Additional question for water consumption

Total amount of water consumed ☐☐☐☐ ml in a day

References

Almeida, O. P. and Almeida, S. A. (1999) Short versions of the Geriatric Depression Scale: A study of their validity for the diagnosis of a major depressive episode according to ICD-10 and DSM-IV. *International Journal of Geriatric Psychiatry*, 14, 858–865.

Eaton, W. W., Muntaner, C., Smith, C., Tien, A. and Ybarra, M. (2004) Center for Epidemiologic Studies Depression Scale: Review and Revision (CESD and CESDR). in *The use of psychological testing for treatment planning and outcomes assessment. Vol 3: Instruments for adults*.

Guralnik, J. M., Simonsick, E. M., Ferrucci, L., Glynn, R. J., Berkman, L. F., Blazer, D. G., Scherr, P. A. and Wallace, R. B. (1994) Aassessing lower extremity function: Association with self-reported disability and prediction of mortality and nursing home admission. *Journals of Gerontology*.

Kaiser, M. J., Bauer, J. M., Ramsch, C., Uter, W., Guigoz, Y., Cederholm, T., Thomas, D. R., Anthony, P., Charlton, K. E., Maggio, M., Tsai, A. C., Grathwohl, D., Vellas, B. and Sieber, C. C. (2009) Validation of the Mini Nutritional Assessment short-form (MNA®-SF): A practical tool for identification of nutritional status. *Journal of Nutrition, Health and Aging*.

Katz, S. (1983) Assessing self-maintenance: activities of daily living, mobility, and instrumental activities of daily living. *Journal of the American Geriatrics Society*. United States, 31, 721–727.

National Institute for Health Research Southampton Biomedical Research Centre (2015). *Procedure for Measuring Adult Circumferences.* https://www.uhs.nhs.uk/Media/Southampton-Clinical-Research/Procedures/BRCProcedures/Procedure-for-adult-circumference-measurements.pdf

National Library of Medicine (2017). *Height – Standing Height.* https:// https://cde.nlm.nih.gov/formView?tinyId=mkugwsfp3ie

Radloff, L. S. (1977a) The CES-D Scale: A Self-Report Depression Scale for Research in the General Population. *Applied Psychological Measurement*.

Radloff, L. S. (1977b) The CES-D Scale. *Applied Psychological Measurement*.

Rubenstein, L. Z., Harker, J. O., Salva, A., Guigoz, Y. and Vellas, B. (2001) Screening for Undernutrition in Geriatric Practice: Developing the Short-Form Mini-Nutritional Assessment (MNA-SF). *The Journals of Gerontology Series A: Biological Sciences and Medical Sciences*, 56, M366–M372.

Stone, L. E., Granier, K. L. and Segal, D. L. (2019) Geriatric Depression Scale. in *Encyclopedia of Gerontology and Population Aging*. Cham: Springer International Publishing, 1–8.

Taylor, H. L., Jacobs, D. R., Schucker, B., Knudsen, J., Leon, A. S. and Debacker, G. (1978) A questionnaire for the assessment of leisure time physical activities. *Journal of Chronic Diseases*, 31, 741–755.

The Tufts University Nutrition Collaborative (2017). *Hand Grip Strength Protocol*. https://cdaar.tufts.edu/protocols/Handgrip.pdf

The Tufts University Nutrition Collaborative (2017). *Bioelectrical Impedence Analysis (BIA) Protocol*. https://cdaar.tufts.edu/protocols/BIA2.pdf

Vellas, B., Guigoz, Y., Garry, P. J., Nourhashemi, F., Bennahum, D., Lauque, S. and Albarede, J.-L. (1999) The mini nutritional assessment (MNA) and its use in grading the nutritional state of elderly patients. *Nutrition*, 15, 116–122.

Yesavage, J. A. (1988) Geriatric Depression Scale (Short Version). *Psychopharmacology Bulletin*.

Yesavage, J. A., Brink, T. L., Rose, T. L., Lum, O., Huang, V., Adey, M. and Leirer, V. O. (1982) Development and validation of a geriatric depression screening scale: A preliminary report. *Journal of Psychiatric Research*.

Yesavage, J. A. and Sheikh, J. I. (1986) 9/Geriatric Depression Scale (GDS). *Clinical Gerontologist*, 5, 165–173.
